# Identification of a Negative Regulator for Salt Tolerance at Seedling Stage via a Genome-Wide Association Study of Thai Rice Populations

**DOI:** 10.3390/ijms23031842

**Published:** 2022-02-06

**Authors:** Thammaporn Kojonna, Thiti Suttiyut, Nopphakhun Khunpolwattana, Monnat Pongpanich, Duangjai Suriya-arunroj, Luca Comai, Teerapong Buaboocha, Supachitra Chadchawan

**Affiliations:** 1 Biotechnology Program, Faculty of Science, Chulalongkorn University, Bangkok 10330, Thailand; thammaporn.ko@gmail.com; 2 Center of Excellence in Environment and Plant Physiology (CEEPP), Department of Botany, Faculty of Science, Chulalongkorn University, Bangkok 10330, Thailand; tsuttiyu@purdue.edu (T.S.); nopphakhun.kh@gmail.com (N.K.); 3 Department of Mathematics and Computer Science, Faculty of Science, Chulalongkorn University, Bangkok 10330, Thailand; monnat.p@chula.ac.th; 4 Omics Sciences and Bioinformatics Center, Faculty of Science, Chulalongkorn University, Bangkok 10330, Thailand; teerapong.b@chula.ac.th; 5 Rice Department, Ministry of Agriculture and Cooperatives, Bangkok 10900, Thailand; duangjai.s@rice.mail.go.th; 6 Department of Plant Biology and Genome Center, University of California Davis, Davis, CA 95616, USA; lcomai@ucdavis.edu; 7 Molecular Crop Research Unit, Department of Biochemistry, Faculty of Science, Chulalongkorn University, Bangkok 10330, Thailand

**Keywords:** genome-wide association study (GWAS), salt response, rice, seedling stage, *OsCRN*

## Abstract

Salt stress is a major limiting factor in crop production and yield in many regions of the world. The objective of this study was to identify the genes responsible for salt tolerance in Thai rice populations. We performed a genome-wide association study with growth traits, relative water content, and cell membrane stability at the seedling stage, and predicted 25 putative genes. Eleven of them were located within previously reported salt-tolerant QTLs (ST-QTLs). *OsCRN*, located outside the ST-QTLs, was selected for gene characterization using the *Arabidopsis* mutant line with T-DNA insertion in the orthologous gene. Mutations in the *AtCRN* gene led to the enhancement of salt tolerance by increasing the ability to maintain photosynthetic pigment content and relative water content, while the complemented lines with ectopic expression of *OsCRN* showed more susceptibility to salt stress detected by photosynthesis performance. Moreover, the salt-tolerant rice varieties showed lower expression of this gene than the susceptible rice varieties under salt stress conditions. The study concludes that by acting as a negative regulator, OsCRN plays an important role in salt tolerance in rice.

## 1. Introduction

Rice is a staple food for more than half of the world’s population. It is grown in more than a hundred countries; however, rice production has been lower than the consumption demand due to the rapidly growing population and the limited water availability [1]. Salt stress is a major limiting factor responsible for reduced crop yield and productivity in many regions of the world [2]. Salinity tolerance is a complex trait whose expression depends on the action and interaction of different morphological, physiological, and biochemical characteristics of plants, including growth, photosynthesis, and grain yield [3,4].

Genome-wide association studies (GWAS) based on single nucleotide polymorphism (SNP) markers have been widely used in rice and other plants [5]. Huang et al. [6] successfully performed GWAS in a rice landrace collection in China for 14 agronomic traits and identified a substantial number of loci with high resolution. A total of 950 rice varieties were used to apply GWAS to discover the associate loci underlying flowering time and grain yield traits, and 32 novel loci were identified [7]. Recently, GWAS has been used to determine the loci with salt tolerance in rice [8,9,10,11,12,13]. Kong et al. (2021) [8] identified LOC_Os06g01250 and LOC_Os06g37300, both of which encode cytochrome P450, and LOC_Os05g14880, encoding proline-rich family protein as candidate genes for salt tolerance, while Nayyeripasand et al. (2021) [9] reported several candidate genes, such as the genes encoding cation chloride cotransporter, WRKY transcription factor (WRKY 12), a transcriptional activator of α-amylase, and high affinity K^+^ transporter (HAK). Moreover, GWAS has also been carried out in other crops such as barley [14,15], wheat [16,17], sorghum [18], lettuce [19] maize [20,21], and *Arabidopsis* [22].

Thailand has more than 17,000 local rice varieties, conserved at the National Rice Gene Bank of Thailand and these are valuable genetic resources for GWAS [23]. The SNP data obtained from local Thai rice populations can be used to identify the causative genes for the traits of interest. Lekklar et al. [24] performed GWAS to identify salinity-responsive genes at the flowering stage of the rice population from Thailand and reported 73% of the identified loci located with previously reported salt tolerance QTLs. Therefore, this study aimed to use GWAS to predict salt-responsive genes in local Thai rice populations at the seedling stage and to understand the function of genes by studying homolog genes in *Arabidopsis* mutant lines.

## 2. Results

### 2.1. Phenotypic Variation in Local Thai Rice Seedlings under Salinity Stress

The average values of growth parameters, relative water content, and cell membrane stability are shown in Table 1. There was variation for all phenotypic traits under both control and salt stress conditions in local Thai rice population. To determine the effect of genotypic variation on salt-stress responses, the salt stability index and percentage change of each rice accession were calculated (Supplementary data Tables S1–S3). The local Thai rice cultivars in this experiment showed different salt-stress responses, presumably consistent with the level of salt tolerance of each cultivar. The sensitive cultivars displayed a low stability index, while the more salt-tolerant cultivars had a stability index close to 1 or more. A stability index higher than 1 indicated that salinity conditions in soil at EC 9–10 dSm^−1^ could enhance the growth of certain varieties. Examples of these varieties were ‘Pratahn Ban Bung’, ‘Leuang Puang Tawng’, and ‘Khiaw Hahng Mah’ (Supplementary Data, Tables S1 and S2).

**Figure 1:**
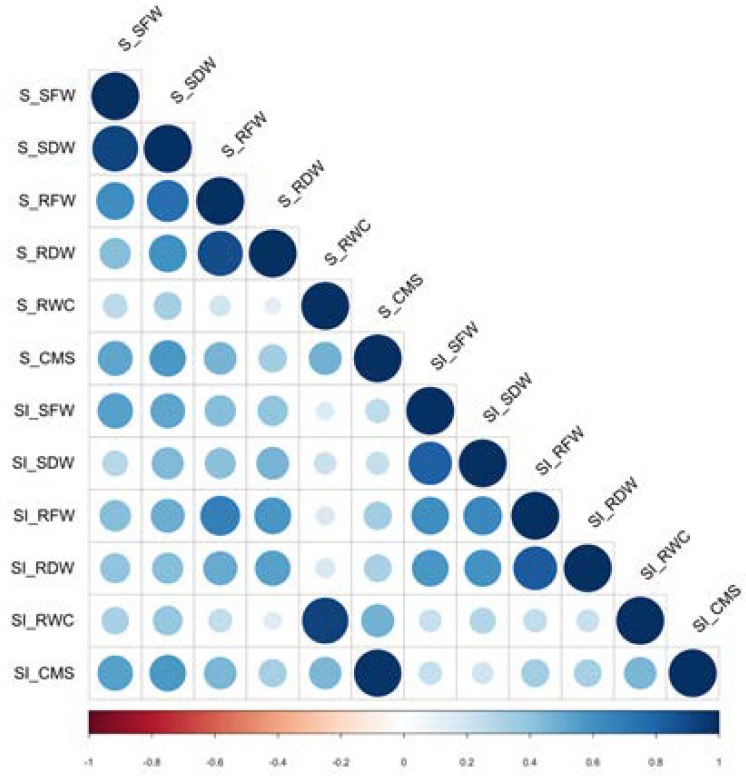
Correlations among phenotypic traits, such as shoot fresh weight (S_SFW), shoot dry weight (S_SDW), root fresh weight (S_RFW), root dry weight (S_RDW), relative water content (S_RWC), and cell membrane stability (S_CMS) under salt stress condition, including the stability index (SI).

Correlation coefficients of all phenotypic traits and their stability indices were calculated (Figure 1). There were positive correlations between biomass parameters (SFW, SDW, RFW, and RDW), cell membrane stability (CMS), and relative water content (RWC). RWC under salt stress (S_RWC) showed a low correlation (0.2–0.4) with growth parameters obtained from the same plants, while CMS under salt stress (S_CMS) had a higher correlation with shoot growth (S_SFW and S_SDW) than with root growth (S_RFW and S_RDW). Interestingly, the stability index of RWC (SI-RWC) was correlated with RWC under salt stress (S_RWC), and the stability index of CMS (SI-CMS) was highly correlated with CMS under salt stress (S_CMS), suggesting that RWC and CMS under salt stress conditions, without comparison with the value under normal-grown conditions, are appropriate for determining salt tolerance in rice.

### 2.2. Genome-Wide Association between Phenotypic Traits under Salt Stress and SNPs

**Figure 2:**
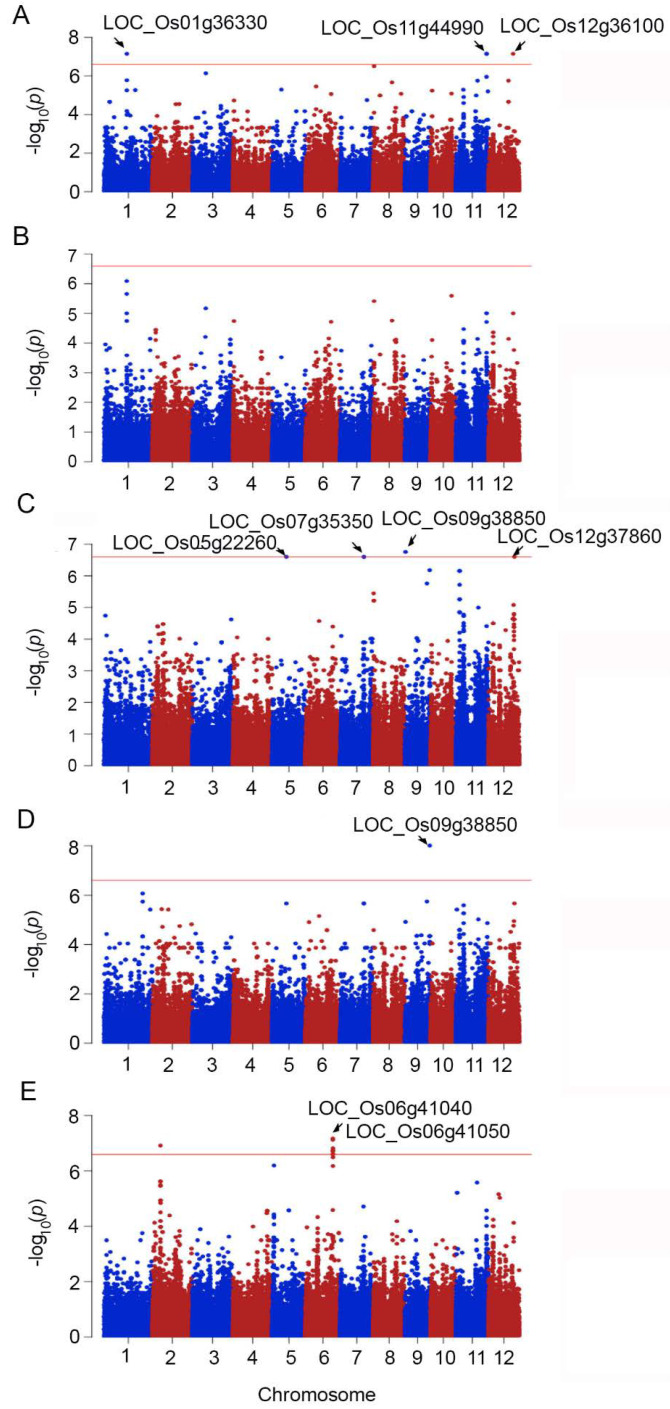
Manhattan plots demonstrating the genome-wide association between SNPs and salt responsive phenotypes based on shoot fresh weight (SFW) (**A**), shoot dry weight (SDW) (**B**), root fresh weight (RFW) (**C**), root dry weight (RDW) (**D**), and cell membrane stability (CMS) (**E**). Some loci with significant SNPs were indicated in Manhattan plots.

The average values of shoot fresh weight, shoot dry weight, root fresh weight, root dry weight, and cell membrane stability under salt stress conditions were used to perform a genome-wide association study based on SNP markers obtained from exome sequencing. GWAS of SFW revealed significant SNPs in three loci, *LOC_Os01g36630*, *LOC_Os11g44990*, and *LOC_Os12g36100*, which encode the expressed proteins, NB-ARC domain-containing protein, and kinesin-4, respectively (Figure 2A). The association of SDW and SNPs in the exomes did not result in significant causative SNPs (Figure 2B). Four causative genes predicted for RFW were *LOC_Os05g22260*, *LOC_Os07g35350*, *LOC_Os09g38850*, and *LOC_Os12g37860*, which encoded crooked neck (CRN) protein, glucan endo-1,3-beta-glucosidase precursor, DUF26 kinases, and expressed protein, respectively (Figure 2C). The association between SNPs and RDW exhibited a single causative gene, *LOC_Os09g38850* encoding OsWAK91–OsWAK receptor-like protein kinase (Figure 2D). Two chromosomes, chromosome 2 and chromosome 6, contained causative regions for CMS traits. The predicted region on chromosome 2 was not associated with any annotated genes, whereas SNPs were located in a gene cluster consisting of *LOC_Os06g41040, LOC_Os06g41050, LOC_Os06g41060,* and *LOC_Os06g41110*. The details of these genes are listed in Table 2.

**Figure 3:**
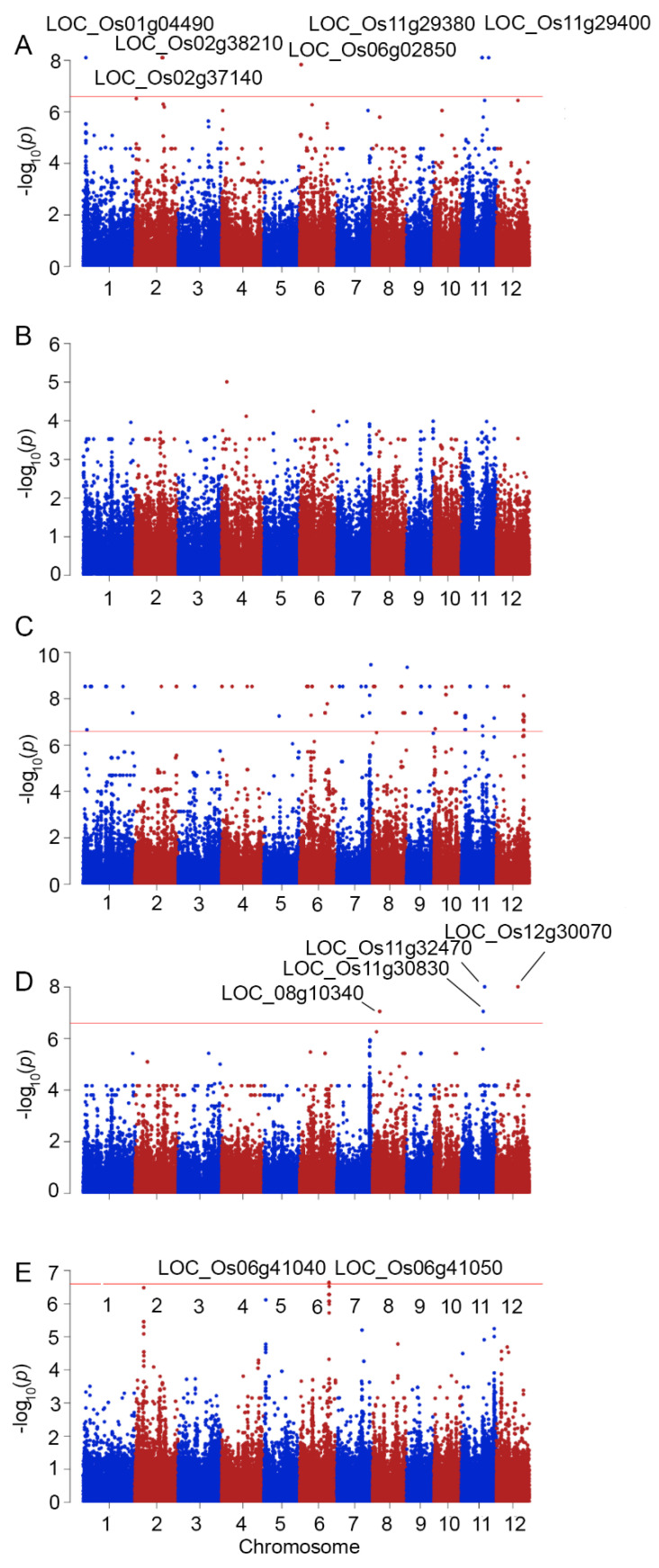
Manhattan plots demonstrating the genome-wide association between SNPs and stability index of salt responsive phenotypes based on shoot fresh weight (SI_SFW) (**A**), shoot dry weight (SI_SDW) (**B**), root fresh weight (SI_RFW) (**C**), root dry weight (SI_RDW) (**D**), and cell membrane stability (SI_CMS) (**E**). Some loci with significant SNPs are indicated in Manhattan plots.

The stability indices of SFW, SDW, RFW, RDW, and CMS were associated with SNPs in exomes, revealing the causative genes in the salt-tolerant response, as shown in Figure 3 and Table 2. The association with stability indices resulted in the different patterns of qq-plots (Supplementary Figures S1 and S2) when compared to GWAS performed with the phenotypic traits of the salt-stressed plants, which reflected more causative SNPs. However, these may contain more false-positive results. A large number of false positive signals were also detected in the GWAS using RWC from salt-stressed plants and SI_RWC (data not shown).

GWAS revealed different causative regions associated with SFW. Six regions were located on chromosomes 1, 2, 6, and 11 (Figure 3A). This was different from the GWAS of SI_SDW, which revealed no significant SNPs (Figure 3B). GWAS of SI_RFW resulted in more than 40 significant SNPs and with a pattern of qq-plot, which may contain a number of false positive predictions (Figure 3C). GWAS for SI_RDW revealed four positive SNPs located on chromosomes 8, 11, and 12 (Figure 3D). Two significant SNPs were located on *LOC_Os08g10340*, encoding OsFBX278–F-box domain-containing protein, while the significant SNPs found on chromosome 11 were located in *LOC_Os11g30830* and *LOC_Os11g32470*, encoding the expressed protein and no exine formation 1 (NEF1) gene, respectively. The predicted SNPs on chromosome 12 were located on *LOC_Os12g30070* (Table 2), encoding the disease resistance protein RPM1, whose ortholog in *Arabidopsis* was reported to be responsible for the rapid increase in cytosolic Ca^2+^ required for the oxidative burst during the hypersensitive response, leading to cell death [25]. There were two significant SNPs for SI_CMS at *LOC_Os06g41040* and *LOC_Os06g41050*, which were the same loci detected by GWAS with CMS values obtained from plants under salt stress (Figure 3E, Table 2).

**Figure 4:**
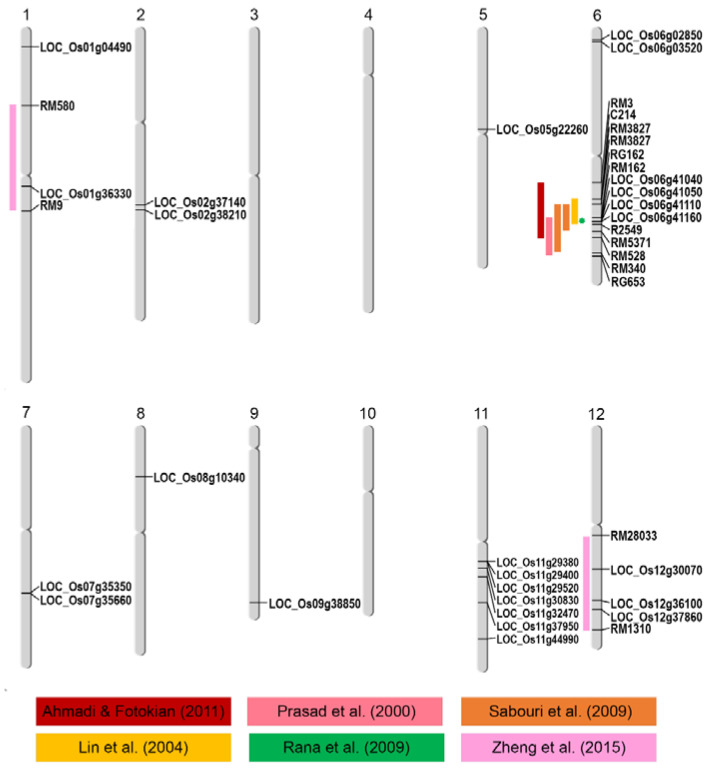
The location of the causative genes predicted by GWAS in a Thai rice seedling population. For comparison, known salt-tolerant quantitative trait loci (ST-QTLs) are indicated by the colored bands on the left of the chromosomes with the associated chromosomal markers.

Based on GWAS with various parameters obtained from salt-stressed rice seedlings, a total of 25 causative genes were predicted. These were located on chromosomes 1, 2, 5, 6, 7, 8, 9, 11, and 12. The map of all predicted genes in comparison with the reported salt-tolerant QTLs (ST-QTLs) is shown in Figure 4.

### 2.3. Characterization of a Gene Putative Associated with Salt Tolerance Using an Arabidopsis Mutant 

*LOC_Os05g22260* (*OsCRN*) was selected for further characterization of salt tolerance based on its interaction with other proteins predicted by the STRING database (Supplementary Figure S3), especially the *AtSKIP* gene, which has been reported to confer osmotic tolerance during salt stress [26]. *AtCRN* (AT5G41770) is a homologous gene of *OsCRN* in *Arabidopsis*. Therefore, an *Arabidopsis* mutant line with T-DNA insertion at the *AtCRN* gene, a *crn* mutant, from the *Arabidopsis* Biological Resource Center (ABRC), was selected to study the role of the *AtCRN* gene in salt tolerance.

**Figure 5:**
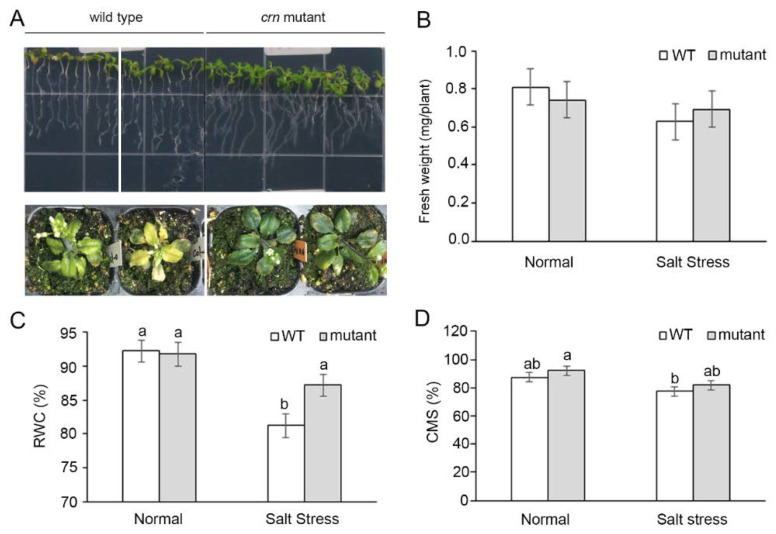
The phenotypes of *crn* mutant compared with wild type (WT). Seven-day-old seedlings were grown in MS medium supplemented with 100 mM NaCl for 7 days or 4-week-old soil-grown plants supplemented with 350 mM NaCl for 6 days (**A**). Fresh weight (**B**), relative water content (RWC) percentage (6 days after salt stress treatment) (**C**), and cell membrane stability (CMS) (9 days after salt stress treatment) (**D**) were compared between plants grown in the normal condition and salt stress (350 mM NaCl) condition. Error bars represent standard error. The different letters above the bar graph indicate the significant difference between means by DMRT analysis at *p* < 0.05.

When 7-day-old seedings were planted on MS medium supplemented with 100 mM NaCl for 7 days, there was no significant difference in biomass between the wild-type (WT) and mutant lines (Figure 5A). Therefore, we monitored the effects of salt stress on the soil-grown plants. When flower buds emerged (4- to 5-week-old plants), 350 mM NaCl was applied as salt stress treatment, while water was applied to the normal grown plants. After 6 to 9 days, salt stress caused fresh weight reduction in both the WT and *crn* mutants. However, no significant differences were detected (Figure 5B). Salt stress caused a significant reduction in the RWC of WT, but less reduction was detected in the mutant line (Figure 5C). Salt stress also caused a reduction in the CMS. However, there was no significant difference between the WT and mutant strains (Figure 5D). It is worth mentioning that CMS reduction occurred after 9 days of salt stress, while a reduction in RWC could be detected after 6 days of salt stress.

**Figure 6:**
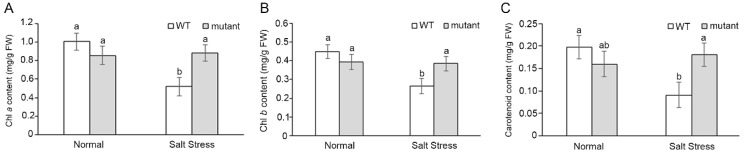
Photosynthetic pigment content, Chl *a* (**A**), Chl *b* (**B**), and carotenoid (**C**) in 4-week-old soil-grown WT and *crn* mutant, treated for 6 days. Error bars represent standard error. The different letters above the bar graph indicate the significant difference between mean by DMRT analysis at *p* < 0.05.

The *crn* mutant had the ability to maintain photosynthetic pigment content under salt stress conditions (Figure 5A and Figure 6). Salt stress caused photosynthetic pigment degradation in WT plants after 6 days under stress. In WT, Chl *a* (Figure 6A), Chl *b* (Figure 6B), and carotenoid were decreased by 48%, 41%, and 54%, respectively, while in the mutant line, a slight reduction in Chl *b* (2%) and a slight increase in Chl *a* (4%) (Figure 6A) and carotenoid (14%) (Figure 6C) were detected. However, there was no significant difference in photosynthetic pigment content between the salt-stressed and non-stressed *crn* mutant (Figure 6).

### 2.4. Ectopic Expression of the OsCRN Gene in crn Mutant and WT Increased the Susceptibility to Salt Stress

In order to validate *OsCRN* gene involvement in the salt tolerance response, it was expressed in the *crn Arabidopsis* mutant and WT. Two complemented lines, rev-B and rev-D, and two ectopic expression lines, Ox-R and Ox-L, were generated. After the screening of homozygous plants in the T_2_ generation, the homozygous lines were used to evaluate the photosynthetic responses under normal and salt stress conditions, as the *crn* mutant line showed the ability to maintain photosynthetic pigments. Therefore, evaluation of the photosynthetic response is a sensitive method for monitoring the response of these lines under salt stress.

**Figure 7:**
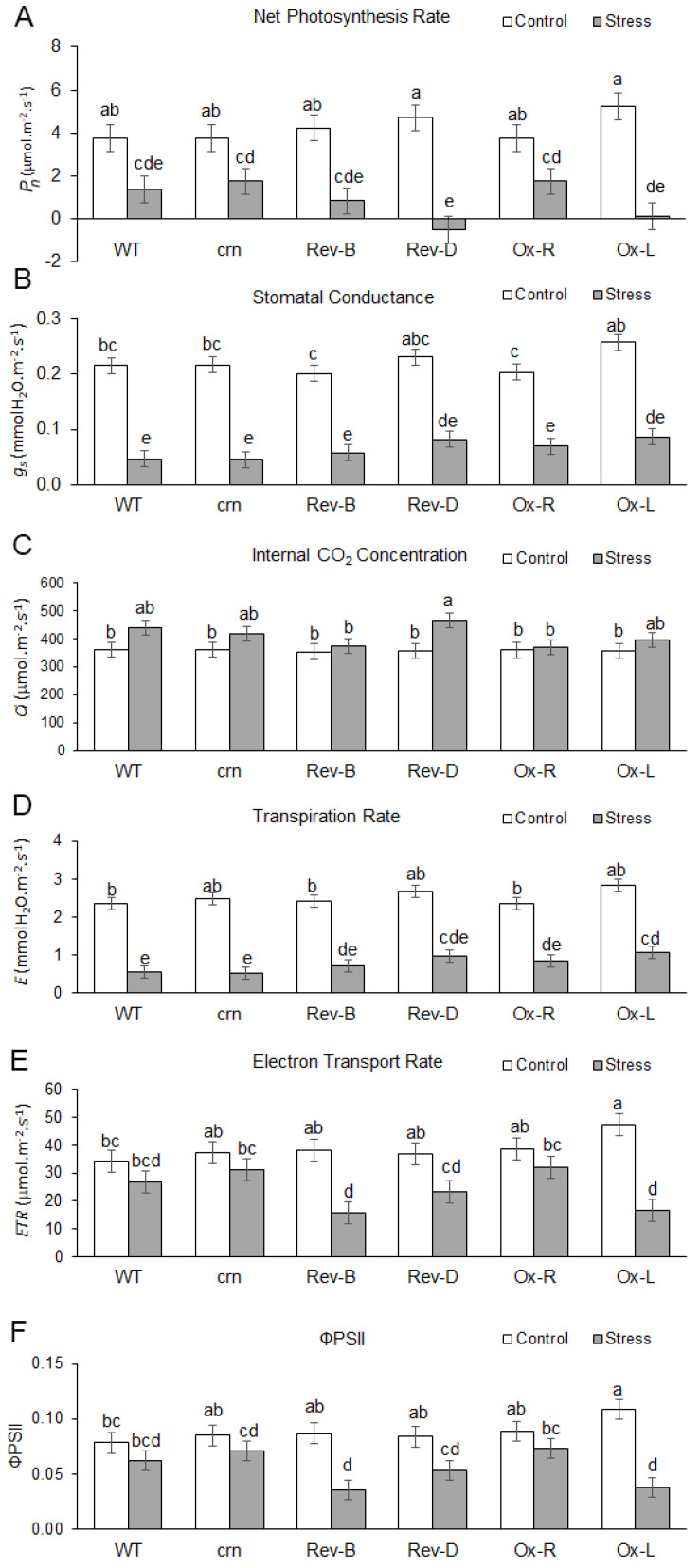
Photosynthesis performance of WT, *crn* mutant, the complemented lines by the expression of *OsCRN* gene, Rev-B and Rev-D, and the ectopic expression line of *OsCRN* gene in the WT genetic background, Ox-R and Ox-L. The photosynthesis performance is shown with the net photosynthesis rate or *P_n_* (**A**), stomatal conductance or *g_s_* (**B**), internal CO_2_ concentration or *C_i_* (**C**), transpiration rate or *E* (**D**), electron transport rate or *ETR* (**E**), and quantum yield of PSII or φPSII (**F**). The different letters above the bar graph indicate the significant difference between mean by DMRT analysis at *p* < 0.05.

Four-week-old plants were treated with 350 mM NaCl for salt stress and plain water was added under normal conditions. At the beginning of the experiment, no significant differences were found in the photosynthetic parameters among these lines. After 7 days, salt stress decreased net photosynthesis rate (*P_n_*) in all lines (Figure 7A). The *P_n_* of the *crn* mutant was slightly higher than that of the WT, while the *P_n_* of one of the complemented lines, Rev-D, was less than 0, resulting in a significant difference from the *crn* mutant. The *P_n_* of Rev-B and Ox-L was also lower than that of the *crn* mutant (Figure 7A). A significant difference in stomatal conductance (*g_s_*) was not detected in salt-stressed plants. However, *crn* mutants tended to have lower *g_s_* than WT, while *g_s_* of the complemented lines and ectopic expression lines tended to have higher *g_s_* than the *crn* mutant. Salt stress caused an increase in internal CO_2_ concentration (*C_i_*) in all lines. The highest *C_i_* was detected in Rev-D, which was consistent with the greatest decline in the *A* (Figure 7C). The transpiration rate (*E*) was consistent with that of *g_s_*. Rev-D and Ox-L had the highest transpiration rate (Figure 7D).

Expression of *OsCRN* inhibited electron transport in photosynthesis and decreased the quantum yield of φPSII. In general, salt stress decreased the electron transport rate (ETR) and quantum yield of φPSII. This phenomenon was observed in all lines. However, the ETRs of Rev-B, Rev-D, and Ox-L were significantly lower than their respective ETRs under normal growth conditions, suggesting a greater susceptibility to salt stress in these lines. Only a 16% reduction in ETR was detected in the *crn* mutant, whereas WT had a 21% reduction in ETR. In contrast, a 59% and 37% decrease in ETR was found in Rev-B and Rev-D, respectively, while 17% and 65% of ETR reduction was found in the ectopic expression lines, Ox-R and Ox-L (Figure 7E). 

The quantum yield of photosystem II (φPSII) was investigated under normal and salt-stressed conditions. The ectopic expression of Ox-L had significantly higher φPSII than WT under normal growth conditions, while other lines showed similar levels of φPSII under normal conditions. Salt stress caused a decline in φPSII in all the lines. Ox-L was the most susceptible to salt stress, with the highest reduction in φPSII, while the *crn* mutant had the least reduction in φPSII (Figure 7F).

### 2.5. The Salt-Tolerant Rice Varieties Lowered OsCRN Gene Expression under Salt Stress Condition

**Figure 8:**
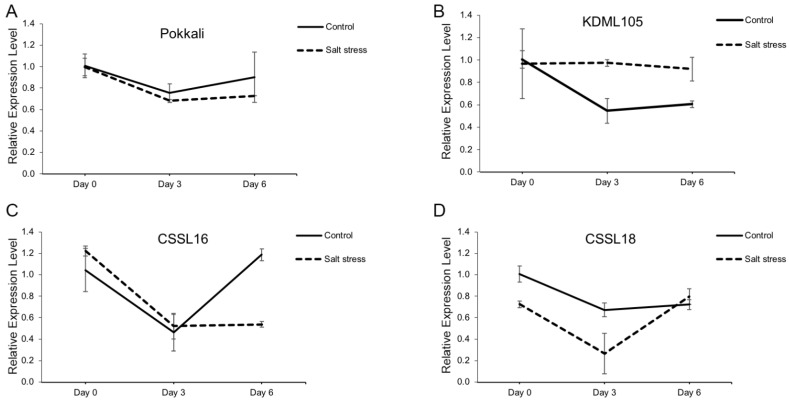
*OsCRN* gene expression in salt-tolerant standard cultivar, ‘Pokkali’ (**A**), Thai elite cultivar (more susceptible to salt stress), ‘KDML105’ (**B**), and the two chromosome substitution lines with ‘KDML105’ genetic background, CSSL16 (**C**) and CSSL18 (**D**). OsEF1α was used as the internal control.

*OsCRN* gene expression was investigated in four rice varieties, namely the salt-tolerant rice variety ’Pokkali’, the salt-susceptible Thai rice variety ‘KDML105’, and two salt-tolerant lines with ‘KDML105’ genetic background, CSSL16 [27,28,29] and CSSL18 [30]. A similar level of *OsCRN* gene expression was detected in ‘Pokkali’ when it was grown in normal (control) and salt stress condition (Figure 8A), while the opposite pattern was found in ‘KDML105’. Under salt stress, ‘KDML105’ had a higher level of *OsCRN* gene expression than that observed under normal conditions (Figure 8B). On the other hand, in the CSSL16, *OsCRN* gene expression was lower than in the normal grown plants after 6 days of treatment (Figure 8C), while *OsCRN* expression of CSSL18 was lower than that in normal grown plants after 3 days of treatment (Figure 8D). These results support the role of *OsCRN* as a negative regulator of salt tolerance in rice.

## 3. Discussion

Growth parameters, such as SRW, SDW, RFW, and RDW, have been used to determine salt stress responses and tolerance in various species, such as cotton [31], maize [32,33], tomato [34], wheat [35], eggplant [36], and rice [9]. RWC and CMS have also been used to assess salt tolerance. CMS was used to screen for salt-tolerant wheat. CMS was shown to have a positive correlation with Na^+^ and a negative correlation with K^+^ and grain yield. However, the correlation between CMS and RWC depended on the variety tested [37]. In our study, the correlation between CMS and shoot weight (SFW and SDW) was higher than the correlation between RWC and shoot weight. A moderate correlation between CMS and RWC was found, suggesting that different genes contribute to these traits in rice. This was consistent with the GWAS results showing that the regions predicted by GWAS of the growth traits were different from the genes/regions predicted by CMS and RWC.

The genes predicted by GWAS were consistent with the previously reported ST-QTLs. Eight genes predicted by GWAS on chromosomes 1, 6, and 12 were located within the previously reported ST-QTLs [38] (Figure 4). The functions of some predicted candidate genes were characterized. *LOC_Os01g59560* encodes OsRLCK46 protein, which is a protein in the superfamily of receptor-like kinases (RLKs). RLK is a transmembrane protein with an extracellular receptor domain and an intracellular kinase domain that perceives and sends signals [39]. Some RLCKs play a role in the development and stress responses in plants. *AtCRCK1* responds to abiotic stresses, including salt stress [40]. Moreover, *OsRLCK46* was down-regulated in 7-day-old seedlings under salt stress conditions [41]. Based on this GWAS analysis, *LOC_Os08g10340*, encoding OsFBX278–F-box domain-containing protein was identified as the candidate gene. An F-box domain-containing protein gene on chromosome 11 was also identified to be involved with salt tolerance in rice [9]. A transcription factor of gibberellin-dependent alpha-amylase (GAMyb) was suggested to regulate carbohydrate metabolism, leading to salt tolerance response. In our GWAS experiment, two candidate genes with the functions in carbohydrate metabolism were predicted, LOC_Os06g41160 and LOC_Os07g35350 (Table 2). This supports the role of carbohydrate metabolism in salt tolerance. The gene co-expression network of ‘Luang Pratahn’ rice, which is one of the local Thai rice cultivars used in this GWAS, also detected the involvement in carbohydrate metabolism via the expression of *OsGAPDH*. This gene was reported to be in the same network with *LOC_Os05g43310* (Photosystem II reaction center W protein) and *LOC_Os10g25030* (red chlorophyll catabolite reductase; *OsRCCR1*), and have a function in chloroplasts [42].

*LOC_Os05g22260* was selected for further characterization. It encodes the OsCRN protein, a putative mRNA splicing factor. The *crn Arabidopsis* mutant line showed a higher salt tolerance phenotype by increasing the ability to maintain RWC, CMS (Figure 5), and photosynthetic pigment content (Figure 6) during salt stress. Since the *crn Arabidopsis* mutant line was able to maintain chlorophyll and carotenoid pigments under salt stress, and previous reports suggested the importance of carbohydrate metabolism balance under abiotic stresses [42,43], we investigated the photosynthesis responses of the mutant, complemented lines, and the ectopically expressed lines to validate the function of *CRN* gene under salt stress. The complemented lines with the expression of *OsCRN*, Rev-B, and Rev-D, including the ectopically expressed line of *OsCRN* in the WT background, exhibited decreased photosynthesis performance (Figure 7A) without the negative effect of stomatal conductance (Figure 7B). This indicated that the reduction in the net photosynthesis rate was not due to stomatal closure. Under salt stress, the ETR (Figure 7E) and φPSII (Figure 7F) of the complemented and ectopic expression lines, Rev-B, Rev-D, and Ox-L, were lower than those of the *crn* mutant. Therefore, under salt stress conditions, *OsCRN* expression affected the light reaction process rather than stomatal responses. This was consistent with GWAS for the Fv’/Fm’ trait under drought stress performed using a rice panel of 221 lines collected from USDA ARS Dale Bumpers National Rice Research Center, Stuttgart, Arkansas, USA [44]. With the negative effect of *OsCRN* gene expression in *Arabidopsis* during salt stress, we propose that *OsCRN* is a negative regulator of salt tolerance.

In order to investigate the relation between *OsCRN* gene expression and salt tolerance in selected rice cultivars, we compared the salt-tolerant standard cultivar, ‘Pokkali’, the salt-susceptible variety, ‘KDML105’, and the latter’s chromosome substitution lines with salt-tolerant phenotypes, CSSL16 [27,28,29] and CSSL18 [30,45]. ‘KDML105’ rice (Figure 8B) and its salt-tolerant CSSLs displayed opposite *OsCRN* expression responses (Figure 8C,D). The decline in *OsCRN* expression after 3–6 days of salt stress is consistent with the salt-tolerant phenotype of CSSL16 at seedling stages [28,29]. For CSSL18, lower *OsCRN* expression during the first 3 days of salt stress may be enough to contribute to salt tolerance. This supports the role of *OsCRN* as a negative regulator in rice.

Reduced expression of *OsCRN* helps explaining CSSL16 salt tolerance at the seedling, tillering, and flowering stages [28,29,46], making it an attractive breeding line for the further development of salt-tolerant rice cultivar. Furthermore, validated genomic regions of local Thai rice cultivars with high stability index under salt stress can contribute to the breeding of salt-tolerant rice.

## 4. Materials and Methods

### 4.1. Plant Materials, Growth Conditions, and Phenotypic Data Collection

The 89 local Thai rice seedlings were germinated in water for 5 days and grown in smectitic clay soil in pots three inches in diameter, supplemented with 13-13-13 osmocote fertilizer. The experiment was conducted in a randomized complete block design (RCBD) with four replicates (one plant/replicate). Due to the large number of rice cultivars, RCBD was used in order to have a similar environment for all cultivars in each block. After that, all 14-day-old seedlings were treated with 115 mM NaCl solution for 6 days (EC 9–10 dSm^−2^, using an EC meter [SevenCompact™ conductivity S230; Metler, USA]). Phenotypic traits, such as shoot fresh weight, shoot dry weight, root fresh weight, root dry weight, relative water content (RWC), and cell membrane stability (CMS) were measured at 0 and 6 days after treatment. 

For the RWC parameter, the youngest fully-expanded leaves were cut into two pieces of approximately 1 cm and weighed for fresh weight (FW). After that, the leaves were soaked in 10 mL of distilled water in a cup closed with a cap at room temperature for 4 h to allow the leaves to reach full hydration; the turgid weight (TW) was then measured. Finally, the leaves were incubated at 60 °C for 3 days and weighed for dry weight (DW). RWC was calculated according to the formula [47]:

(FW-DW)/(TW-DW) × 100.

For CMS, the youngest fully-expanded leaves (100 mg) were chopped into approximately 0.5-cm pieces and soaked in 10 mL of distilled water in a closed centrifuge tube at room temperature for 4 h. The first electrical conductivity (EC_0_) of the sample solutions was measured using an EC meter (SevenCompact™ conductivity S230; Mettler Toledo, USA). Then, the leaf tissue in the centrifuge tube was boiled for 15–30 min. Sample solutions at room temperature was measured for final electrical conductivity value (EC_1_). CMS was calculated according to the formula [48]:

100 − [(EC_0_/EC_1_) × 100].

The stability index was calculated as the salt stress value divided by the control as (Vstress/Vcontrol). 

Percentage change (% change) was calculated as the percentage of difference in value divided by the control value as ((Vstress-Vcontrol)/Vcontrol) × 100.

Correlation test was performed by JMP ver. 9 (SAS Institute Inc., Cary, NC, USA) and figures were generated by the R ‘corrplot’ package. 

### 4.2. Genome-Wide Association Analysis

Association analysis was performed following Lekklar et al. (2019) [24] using genome-wide efficient mixed model association (GEMMA) software [49,50]. The minor/alternate allele frequency (MAF) that was less than 5% was filtered out of SNP data by PLINK 1.07 and left with 197,454 SNPs. The quantile–quantile plot (Q-Q plot) and Manhattan plot in each trait were conducted by R ‘qqman’ package [51]. Manhattan plots were created with chromosome position on the X-axis and -log *p*-value of all SNPs on the Y-axis. Bonferroni correction was used for multiple testing correction and significant SNPs were selected with *p*-values less than 0.05/ total number of SNPs (*p* < 2.53 × 10^−7^).

### 4.3. Validation of Salt Responses in Arabidopsis

#### 4.3.1. Phenotypic Comparison between crn Mutant and Arabidopsis WT

The *crn* mutant line (SALK_030126C) of *Arabidopsis* at the *AT5G41770* gene (*AtCRN1*), which is the orthologous genes of *LOC_Os05g22260,* and wild-type (Col-0) were cultured on MS media, supplemented with 1% sucrose and 0.8% agar in a 120-mm square petri dish under 16/8-h light/dark period at 22 °C with three replications. After 7 days of germination, 20 seedlings of each line were transferred to MS medium (containing the same concentration of sucrose and agar as mentioned above) with or without 100 mM NaCl. After 7 days of treatment, the mutant seedlings were photographed in comparison with the WT.

To evaluate the phenotypes of soil-grown Arabidopsis, seeds were germinated on MS medium, as indicated above. The comparison of the phenotypes was designed in a randomized complete block design (RCBD) with three replications, with five samples per replications in each line. Then, 7-day-old seedlings were transferred to growing medium in 5-cm diameter pots under a 16/8-h light/dark period with 100 mmol.m^−2^. s^−1^ light intensity at 21–23 °C. *Arabidopsis* lines in this experiment took 4–5 weeks after germination until flowering depending on the lines. At first visible flower buds, *Arabidopsis* plants were treated with (salt stress) or without (control) 350 mM NaCl. After 6 days of treatment, fresh weight, relative water content (RWC), and photosynthetic pigment contents were measured, while the cell membrane stability index (CMS) was measured after 9 days of treatment. 

For photosynthetic pigment content measurement, fresh weight of five seedlings were recorded followed by pigment extraction with 80% acetone and storage at 4 °C overnight. The absorbance at 470.0, 646.8, and 663.2 nm was determined using a spectrophotometer (Agilent 8453 UV-visible Spectroscopy System). Chlorophyll *a*, chlorophyll *b*, and carotenoid content were calculated according to Alan [52] using the following formula:

Chlorophyll *a* content = 12.25A_663_ − 2.79A_646_

Chlorophyll *b* content = 21.5A_646_ − 5.1A_663_

Carotenoid content = [(1000A_470_ − 1.82 Chl *a* − 85.02 Chl *b*)] / 198

For RWC, two or three rosette leaves were collected to obtain the minimum fresh weight of 50 mg, and the fresh weight (FW) was determined. Leaves were then placed in 10 mL of distilled water in cups with a lid and kept in the dark at room temperature for 18 h. The turgid weight (TW) was then determined. The leaves were dried at 60 °C for 3 days and weighed for dry weight (DW). The RWC was calculated as (FW-DW)/ (TW-DW) × 100 [47].

For the CMS parameter, two or three rosette leaves from a single plant for one sample were cut and transferred to a centrifuge tube with 10 mL of deionized water in a closed centrifuge tube and shaken overnight at room temperature. The conductivity of the sample solutions (EC_0_) was measured using an EC meter, and cooled sample solutions were measured again after the leaf tissue was autoclaved (EC_1_). CMS was calculated according to the formula [48]:

100 − [(EC_0_/EC_1_) × 100].

#### 4.3.2. Photosynthesis Performance Comparison of Wild Type (WT), crn Mutant, Complemented Lines, Rev-B and Rev-D, and Ectopic Expression Lines, Ox-R and Ox-L

Wild type (Col-0), mutant line (SALK_030126C), two complemented lines (Rev-B and Rev-D), and two overexpression lines (Ox-R and Ox-L) were used to investigate photosynthesis performance. A randomized complete block design (RCBD) was designed with three replications for control and salt stress conditions with two samples per replicate in each line. Individual plants were grown in 7.5-cm diameter pots for ~4–5 weeks under a 16/8-h light/dark period with 100 mmol.m^−2^.s^−1^ light intensity at 21–23 °C. At first visible flower buds, all *Arabidopsis* plants were treated with or without 350 mM NaCl. 

Photosynthetic parameters, net photosynthetic rate (*P_n_*), stomatal conductance (*g_s_*), intercellular CO_2_ concentration (*C_i_*), transpiration rate (*E*), effective quantum yield of PSII photochemistry (ϕ PSII), and electron transport rate (ETR) were determined in the seventh leaf from the base of the plant after 7 days of treatment using LI-6400XT Portable Photosynthesis (Licor Inc., Lincoln, NE, USA) with a 6400-40 fluorometer (Licor Inc., Lincoln, NE, USA) under conditions of saturated light at 1000 µmol m^−2^ s^−1^ with 10% blue light, air CO_2_ concentration (C_a_) at 400 µmol mol^−1^, chamber block temperature at 23 °C, and relative humidity between 55% and 60%.

### 4.4. Vector Construction and Transformation

The full-length cDNA of *OsCRN* from NIAS DNA Bank was amplified using primers containing the restriction sites of *Nco*I and *BstE*II (Supplementary Table S4). The PCR reaction conditions were set according to the manufacturer’s protocol (New England Biolabs, USA). The PCR product was purified using a TIANGEN Universal DNA Purification Kit (Tiangen, China). Both PCR products and pCAMBIA1300 vectors were digested with *Nco*I and *BstE*II according to the manufacturer’s protocol (New England Biolabs) and then ligated together using T4 ligase (New England Biolab) to obtain the expression vector (pCAMBIA1301_OsCRN).

One hundred nanograms of pCAMBIA1301_OsCRN plasmids were transferred to 50 µL *Escherichia coli* DH5α by heat shock transformation [53]. The positive clones were submitted for sequencing to obtain the correct sequences of the construct before use in Agrobacterium transformation.

### 4.5. Agrobacterium Transformation by Freeze Thaw Method

Five hundred nanograms of pCAMBIA1301_OsCRN plasmids were transformed into 50 µL competent *Agrobacterium tumefaciens* cells by the freeze-thaw method according to Höfgen and Willmitzer [54], and selected on LB agar containing 40 µg/mL gentamycin, 20 µg/mL rifampicin, and 50 µg/mL kanamycin. The positive clones were checked for the presence of plasmids by colony PCR.

### 4.6. Construction of Transgenic Arabidopsis and Screening of Homozygous T_3_ Plants

Wild type *Arabidopsis* and the mutant line were used to generate ectopic expression line(s) and complemented line(s) using the floral dipping method [55] with *Agrobacterium tumefaciens* conforming to the pCAMBIA1301_OsCRN plasmid. T_1_ seeds were selected according to the method of Harrison et al. [56]. The existence of the inserted genes was determined using the primers for the hygromycin resistance gene (*HygR*) and *OsCRN,* as shown in Supplementary Table S4. All phenotyping experiments were performed using the homozygous T_3_ plants. 

### 4.7. Gene Expression Analysis of OsCRN under Salt Stress Condition in Rice

#### 4.7.1. Plant Materials and Growth Conditions

Four rice genotypes, ‘Pokkali’, CSSL 16, CSSL 18, and ‘KDML 105′, were grown in WP N0.2 nutrient solution [57]. After germination, 14-day-old seedlings were transplanted into WP N0.2 nutrient solution with and without 75 mM NaCl as salt stress condition and control, respectively. Leaf tissues were collected on days 0, 3, and 6 after treatment for RNA extraction.

#### 4.7.2. RNA Extraction and Gene Expression Analysis

RNA was extracted from leaf tissue using GENEzol™ reagent following the manufacturer’s protocol (Geneaid, Taiwan) and treated with DNase I according to the manufacturer’s protocol (Invitrogen, USA). cDNA synthesis was performed using a cDNA synthesis kit according to the manufacturer’s protocol (Bioneer, Korea). Quantitative RT-PCR was performed using Luna Universal qPCR Master Mix (New England Biolab, USA). The *OsCRN* gene expression in leaf tissues was determined and *OsEF1α* was used as a reference gene; the specific primers are listed in Supplementary Table S4. The expression ratio was calculated according to the method by Pfaffl [58]. The formula is given as:

(1) $$Ratio=\frac{{\left(E_{target}\right)}^{\Delta CP_{target}\left(control-sample\right)}}{{\left(E_{ref}\right)}^{\Delta CP_{ref}\left(control-sample\right)}}$$

### 4.8. Statistical Analysis

Statistical analyses in this work were performed using ANOVA analysis of variance test by IBP SPSS ver. 22 (IBM Corp., Armonk, NY, USA), and the mean values were compared by Duncan’s multiple range test at 95% confidence level.

## 5. Conclusions

We predicted 25 salt-tolerant genes from GWAS of SFW, SDW, RFW, RDW, RWC, and CMS traits in 89 Thai rice cultivars. These genes are located on chromosomes 1, 2, 5, 6, 7, 8, 9, 11, and 12. The *OsCRN* gene, which is involved in the RNA splicing process, was selected to validate its role in salt tolerance. It was demonstrated that the expression of *OsCRN* in either WT *Arabidopsis* or *crn* mutant line led to the effects on light reaction by the decrease in electron transport rate and quantum yield of PSII. Moreover, *OsCRN* gene expression in the salt-tolerant cultivars was lower when the plants were subjected to salt stress, which was not detected in the salt-susceptible cultivars. This suggests the role of *OsCRN* as a negative regulator of salt tolerance in rice.

## Figures and Tables

**Table 1:** Phenotypic values of growth parameters: shoot fresh weight (SFW), shoot dry weight (SDW), root fresh weight (RFW), root dry weight (RDW), relative water content (RWC), and cell membrane stability (CMS) under normal and salt stress conditions. The stability index (SI) and % changes due to salt stress are also shown.

Traits	Normal	Salt Stress	Stability Index	% Change
SFW (g/plant)	1.07 ± 0.47	0.80 ± 0.36	0.77 ± 0.22	−23.43 ± 21.90
SDW (g/plant)	0.15 ± 0.06	0.11 ± 0.04	0.73 ± 0.20	−45.38 ± 19.77
RFW (g/plant)	0.91 ± 0.27	0.48 ± 0.18	0.55 ± 0.20	−27.42 ± 19.67
RDW (g/plant)	0.11 ± 0.04	0.06 ± 0.02	0.53 ± 0.19	−46.55 ± 19.24
RWC (%)	90.48 ± 4.22	83.91 ± 10.48	0.93 ± 0.12	−7.13 ± 11.95
CMS (%)	94.96 ± 2.29	78.40 ± 10.55	0.83 ± 0.11	−17.39 ± 11.40

**Table 2:** List of causative genes obtained from GWAS.

	Trait	SNPs Position	p_Wald	Locus	Gene Name	Description
1	SI_SFW	1999390	7.86 × 10^−9^	LOC_Os01g04490	Ser/Thr protein kinase	protein modification process, kinase activity
2	S_SFW	20146031	7.08 × 10^−8^	LOC_Os01g36330	expressed protein	-
3	SI_SFW	22455127	7.86 × 10^−9^	LOC_Os02g37140	expressed protein	-
4	SI_SFW	23113356	7.86 × 10^−9^	LOC_Os02g38210	elongation factor Tu	translation factor activity and hydrolase activity
5	S_RFW	12601958	2.52 × 10^−7^	LOC_Os05g22260	crooked neck, putative	nucleic acid metabolic process, binding
6	SI_SFW	1048462	1.46 × 10^−8^	LOC_Os06g02850	expressed protein	-
		1048463	1.46 × 10^−8^			
7	SI_SFW	1361687	1.46 × 10^−8^	LOC_Os06g03520	DUF581 domain-containing protein	-
8	S_CMS	24510573	7.54 × 10^−8^	LOC_Os06g41040	pentatricopeptide	-
	SI_CMS	24510573	2.3 × 10^−7^			
9	S_CMS	24516977	6.75 × 10^−8^	LOC_Os06g41050	expressed protein	response to abiotic stimulus, DNA metabolic process, cell cycle, reproduction
	S_CMS	24517076	2.51 × 10^−7^			
	SI_CMS	24516977	2.4 × 10^−7^			
10	S_CMS	24577287	2.51 × 10^−7^	LOC_Os06g41110	tubulin binding cofactor C	involve in the folding and assembly of α- and β-tubulin monomers
11	S_CMS	24628013	2.51 × 10^−7^	LOC_Os06g41160	expressed protein	carbohydrate metabolic process, metabolic process
		24628494	2.51 × 10^−7^			
12	S_RFW	21151187	2.52 × 10^−7^	LOC_Os07g35350	glucan endo-1,3-beta-glucosidase precursor	carbohydrate metabolic process, hydrolase activity
13	S_RFW	21365153	2.52 × 10^−7^	LOC_Os07g35660	DUF26 kinases	kinase activity, protein modification process
14	SI_RDW	6056897	8.8 × 10^−8^	LOC_Os08g10340	OsFBX278–F-box domain-containing protein	-
		6056962	8.8 × 10^−8^			
15	RDW	22317650	9.82 × 10^−9^	LOC_Os09g38850	OsWAK91–OsWAK receptor-like protein kinase	kinase activity, protein binding
16	SI_SFW	17049126	7.86 × 10^−9^	LOC_Os11g29380	MCM2–Putative minichromosome maintenance MCM complex subunit 2	multicellular organismal development and embryo development
17	SI_SFW	17065481	7.86 × 10^−9^	LOC_Os11g29400	6-phosphogluconate dehydrogenase	catalytic activity
18	SI_SFW	17130888	7.86 × 10^−9^	LOC_Os11g29520	NBS-LRR disease resistance protein	protein binding, response to stress
19	SI_RDW	17953322	8.8 × 10^−8^	LOC_Os11g30830	expressed protein	transferase activity, response to endogenous stimulus
20	SI_RDW	19167004	9.7 × 10^−9^	LOC_Os11g32470	NEF1	response to water deprivation
21	SI_SFW	22502331	7.86 × 10^−9^	LOC_Os11g37950	WIP3–Wound-induced protein precursor	protein and carbohydrate binding, response to abiotic and biotic stress
22	SFW	27245040	7.08 × 10^−9^	LOC_Os11g44990	NB-ARC domain-containing protein	protein binding, response to stress
		27245051	7.08 × 10^−8^			
23	SI_RDW	18009787	9.7 × 10^−9^	LOC_Os12g30070	disease resistance protein RPM1	trigger plants defense systems against biotic stress
24	SFW	22127633	7.08 × 10^−8^	LOC_Os12g36100	kinesin-4	nucleotide binding, motor activity
25	RFW	23267837	2.52 × 10^−7^	LOC_Os12g37860	expressed protein	regulation of gene expression, epigenetic

## Data Availability

The authors confirm that data supporting the findings of this study are available in the article and its supplementary materials (Supplementary Tables S1–S4, Supplementary Figures S1–S3). The exome sequence raw data and code used to execute the GWAS are available through direct contact with the corresponding author.

## Supplementary Materials

The following are available online at https://www.mdpi.com/article/10.3390/ijms23031842/s1.
