# Supplementary material for: Identification of a Negative Regulator for Salt Tolerance at Seedling Stage via a Genome-Wide Association Study of Thai Rice Populations
Source: Int J Mol Sci. 2022 Feb 6;23(3):1842. doi: 10.3390/ijms23031842 (PMC8836775; doi:10.3390/ijms23031842)
Supplement: Supplementary file 1 [file ijms-23-01842-s001.zip › ijms-1542512-Supplementary Figure S1.pdf]

A

B

C

D

E

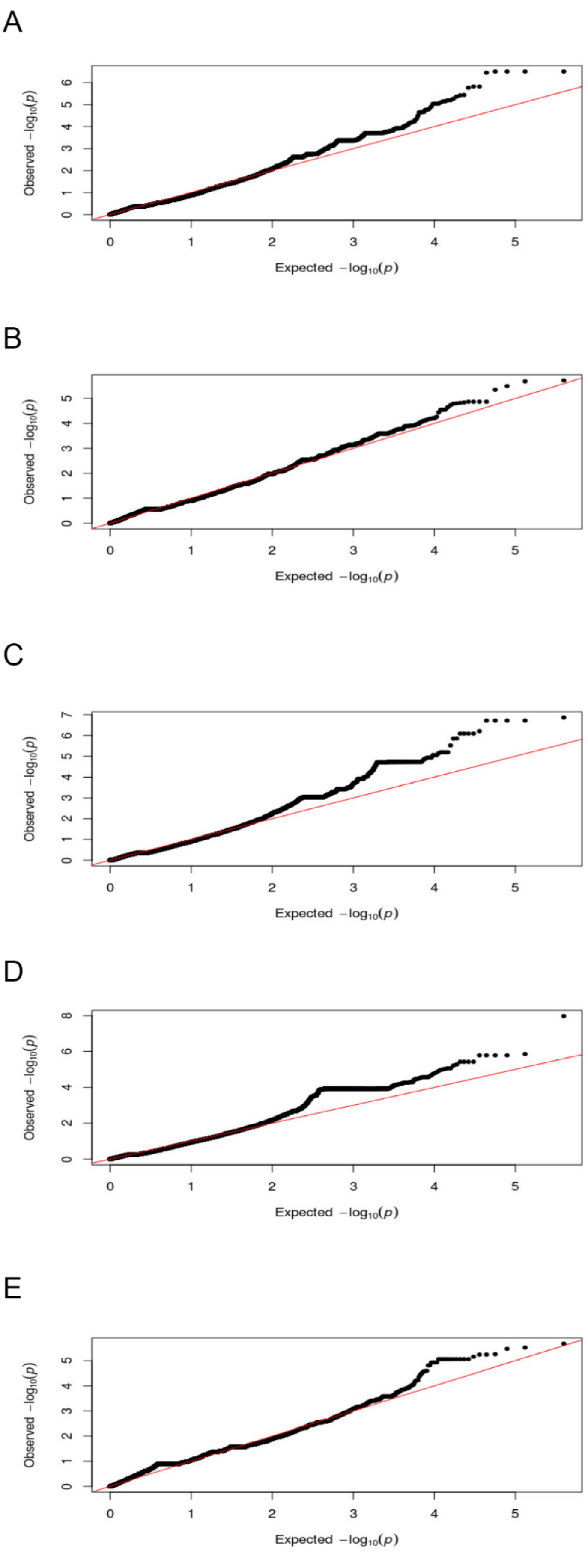

Supplementary Figure S1 QQ plots of the genome-wide association between SNPs of exomes and salt responsive phenotypes, shoot fresh weight (A), shoot dry weight (B), root fresh weight (C), root dry weight (D), and cell membrane stability (E)
