# Supplementary material for: Identification of a Negative Regulator for Salt Tolerance at Seedling Stage via a Genome-Wide Association Study of Thai Rice Populations
Source: Int J Mol Sci. 2022 Feb 6;23(3):1842. doi: 10.3390/ijms23031842 (PMC8836775; doi:10.3390/ijms23031842)
Supplement: Supplementary file 1 [file ijms-23-01842-s001.zip › ijms-1542512-Supplementary Figure S2.pdf]

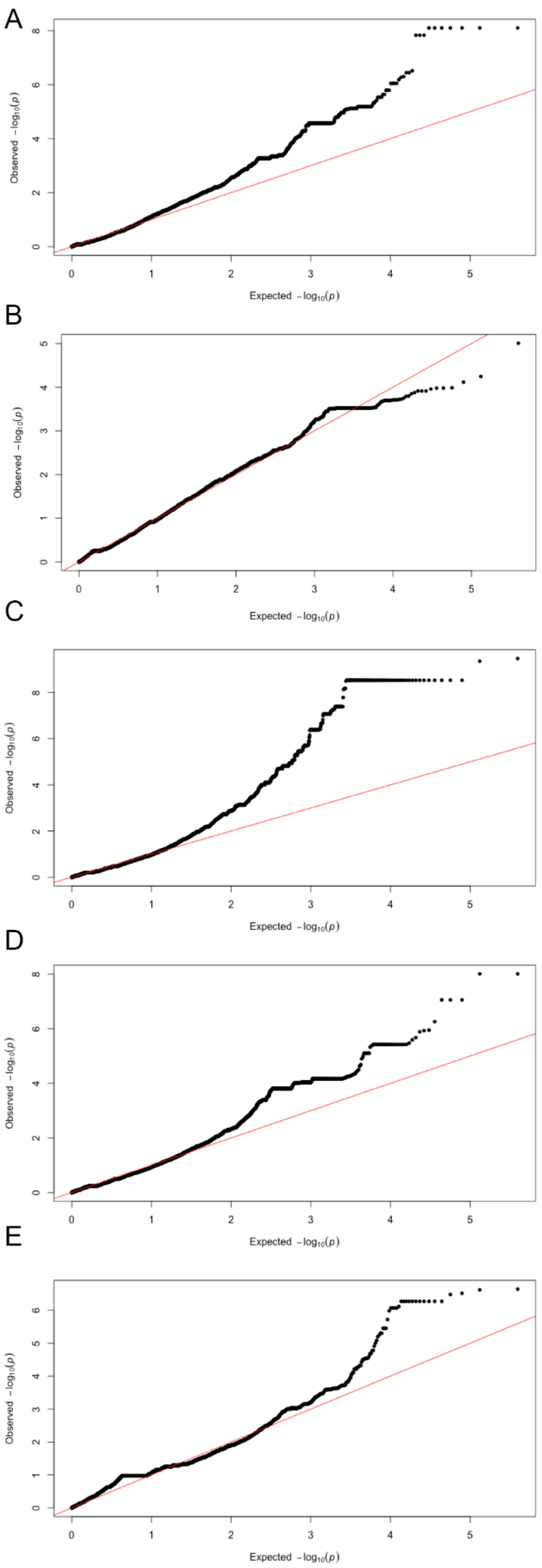

Supplementary Figure S2 QQ plots of the genome-wide association between SNPs of exomes and stability indices of salt responsive phenotypes, shoot fresh weight (SI_SFW) (A), shoot dry weight (SI_SDW) (B), root fresh weight (SI_RFW) (C), root dry weight (SI_RDW) (D), and cell membrane stability (SI_CMS) (E)
