# Supplementary material for: Identification of a Negative Regulator for Salt Tolerance at Seedling Stage via a Genome-Wide Association Study of Thai Rice Populations
Source: Int J Mol Sci. 2022 Feb 6;23(3):1842. doi: 10.3390/ijms23031842 (PMC8836775; doi:10.3390/ijms23031842)
Supplement: Supplementary file 1 [file ijms-23-01842-s001.zip › ijms-1542512-Supplementary Figure S3.pdf]

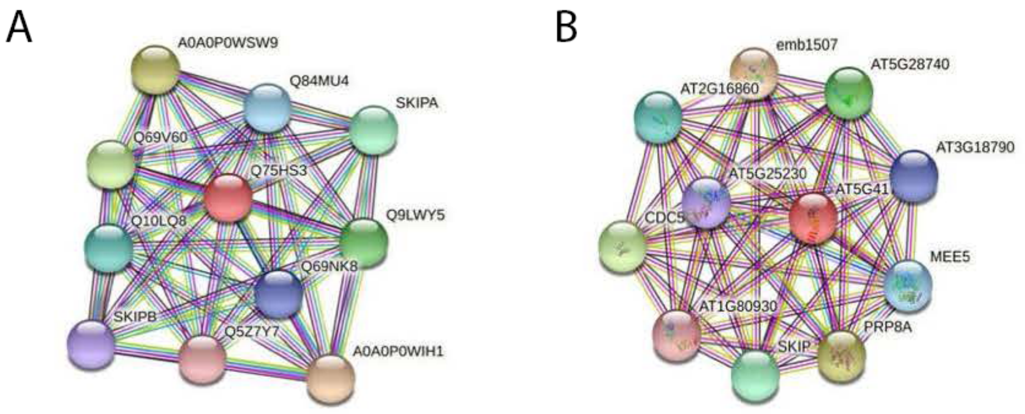

Supplementary Figure S3: Protein-protein interaction between CRN protein and other proteins in rice (A) and *Arabidopsis* (B)
